# Endoplasmic reticulum stress targeted therapy for breast cancer

**DOI:** 10.1186/s12964-022-00964-7

**Published:** 2022-11-07

**Authors:** Di Xu, Zhen Liu, Ming-Xing Liang, Yin-Jiao Fei, Wei Zhang, Yang Wu, Jin-Hai Tang

**Affiliations:** 1 Department of General Surgery, The First Affiliated Hospital of Nanjing Medical University, Nanjing, 210029 People’s Republic of China; 2 Department of Biobank, The First Affiliated Hospital of Nanjing Medical University, Nanjing, 210029 People’s Republic of China

**Keywords:** Breast cancer, Endoplasmic reticulum stress, Unfolded protein response, Signaling pathway, Anticancer drugs

## Abstract

Recurrence, metastasis, and drug resistance are still big challenges in breast cancer therapy. Internal and external stresses have been proven to substantially facilitate breast cancer progression through molecular and systemic mechanisms. For example, endoplasmic reticulum stress (ERS) results in activation of the unfolded protein response (UPR), which are considered an important cellular stress response. More and more reports indicate its key role in protein homeostasis and other diverse functions involved in the process of breast cancer progression. Therefore, therapies targeting the activation of ERS and its downstream signaling pathways are potentially helpful and novel tools to counteract and fight breast cancer. However, recent advances in our understanding of ERS are focused on characterizing and modulating ERS between healthy and disease states, and so little attention has been paid to studying the role and clinical application of targeting ERS in a certain cancer. In this review, we summarize the function and main mechanisms of ERS in different molecular types of breast cancer, and focus on the development of agents targeting ERS to provide new treatment strategies for breast cancer.

**Video Abstract**

**Supplementary Information:**

The online version contains supplementary material available at 10.1186/s12964-022-00964-7.

## Introduction

Breast cancer (BC) is the most common cancer globally and the fifth leading cause of cancer mortality worldwide in women [1]. According to histological expression of three receptor proteins, estrogen receptor (ERα; ESR1), progesterone receptor (PGR), and human epidermal growth factor receptor 2 (ERBB2; HER2; Neu), BC is divided into Luminal A, Luminal B, HER-2 positive, and triple-negative breast cancer (TNBC) [2]. The therapeutic used in treatment depends on the molecular subtypes and tumor stages. Other than surgery, chemotherapy, and radiation therapy, the main therapeutic options include hormone therapy in the Luminal subtypes and molecular targeted therapy in HER-2 positive subtypes [3]. Additionally, immunotherapy has made great progress in the treatment of TNBC [4]. However, therapeutic resistance and metastases are still important factors that frequently lead to treatment failure [2, 5]. Therefore, exploring the underlying mechanisms driving different BC types and identifying novel therapeutic targets may help improve the prognosis of BC patients.

In the progression of BC, cancer cells face extracellular and intracellular stress in the tumor microenvironment. These stress response pathways directly promote the malignant characteristics of tumors, reshape the tumor microenvironment, and weaken anti-tumor immune responses [6]. Among them, endoplasmic reticulum stress (ERS) has been proven to play a key role in cancer development [7]. ER is the organelle for protein secretion and appropriate protein folding to maintain protein homeostasis [8]. In the microenvironment of a malignant tumor, the protein folding ability of the ER in cancer cells and infiltrating immune cells changes, which leads to the accumulation of misfolded and unfolded protein [9]. When the accumulation of misfolded proteins exceeds the tolerable threshold, the three ER sensors will trigger the unfolded protein response (UPR), which results in the activation of a series of complex signaling pathways [10]. Mild ERS can regulate cancer cells and immune cells to promote cancer cell proliferation, metastasis, and drug resistance. Conversely, severe and lethal ERS can trigger immunogenic cell death (ICD) and protective antitumor immunity [7]. Therefore, the results of ERS and the UPR is determined by the duration and intensity of the stress [11].

In this review, we aim to introduce the latest developments of ERS in different types of BC, discuss related functional mechanisms, and illustrate the challenges and likelihood of their therapeutic applications.

## Endoplasmic reticulum stress (ERS) and unfolded protein response (UPR)

**Fig. 1:**
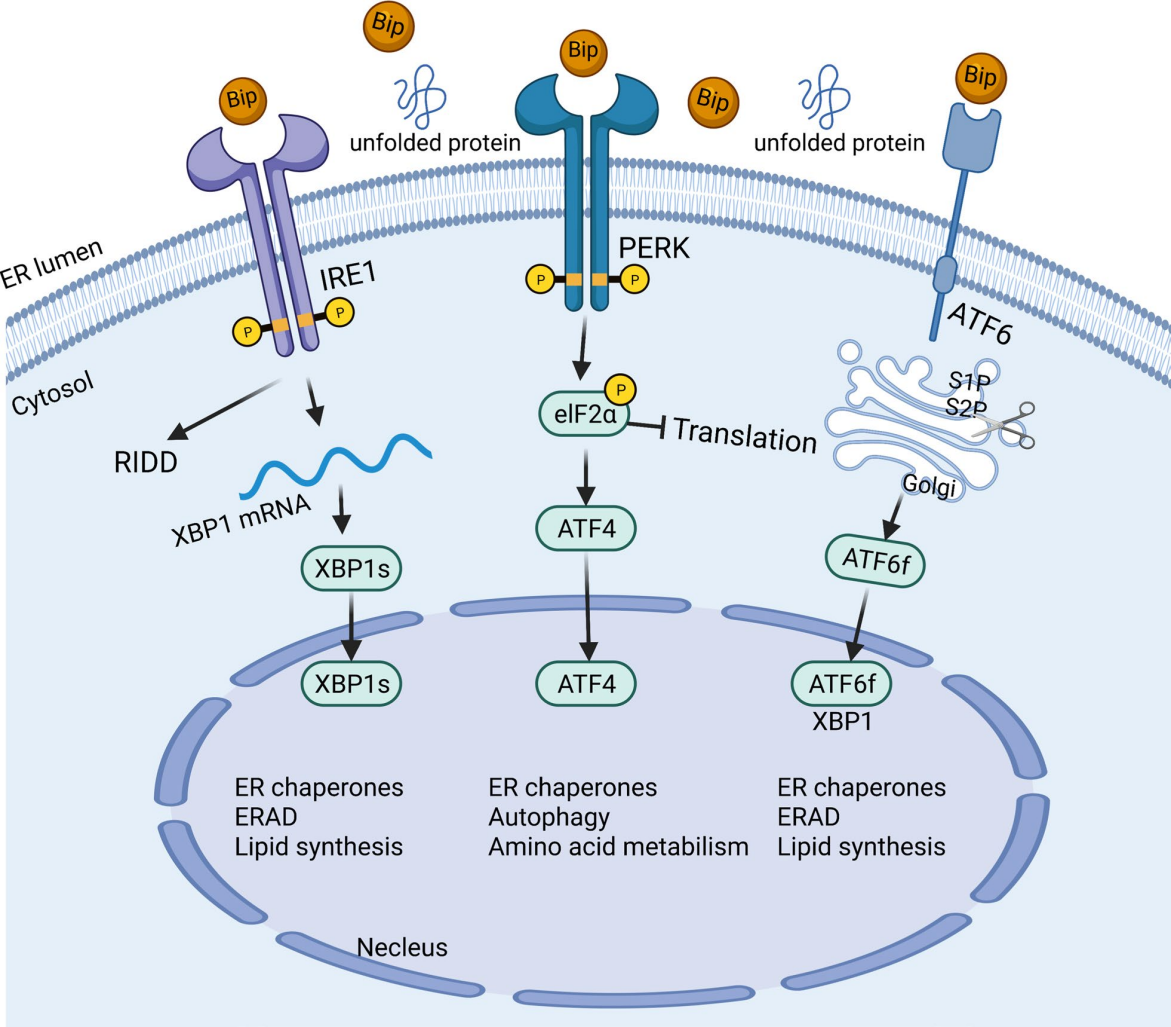
Overview of the three sensors of UPR. Under normal conditions, the three proteins (IRE1a, PERK, and ATF6) bind to the molecular chaperone protein GRP78. While under stress conditions, GRP78 releases from the three sensors, resulting in their activation. Each activation pathway has a different signal transduction mechanism. IRE1α splices XBP1 mRNA to encode for the transcription factor XBP1s, which promotes the expression of genes involved in the protein folding and erase induce and add in ERAD. PERK undergoes oligomerization and auto-phosphorylation which then promotes the phosphorylation of phosphorylate eIF2a, leading to general translational attenuation while selectively activating ATF4. ATF6 is transported from the endoplasmic reticulum to the Golgi apparatus where it undergoes S1P and S2P protease cleavage, which releases the active form of ATF6

ERS and the UPR are regulated by three ER transmembrane proteins: inositol requiring enzyme 1 (IRE1), protein kinase R-like ER kinase (PERK), and activating transcription factor 6 (ATF6) [7]. In cell homeostasis, the endoplasmic reticulum domains of these three proteins bind to molecular chaperone 78-kD glucose-regulated protein (GRP78/BiP). While in ERS conditions, GRP78 is actively recruited to accumulating misfolded proteins and separates from these three proteins (Fig. 1) [12].

ER-localized transmembrane sensor IRE1α and its substrate XBP1 are the most conserved arm of the UPR. After activation, IRE1α undergoes oligomerization and auto-phosphorylation on the cytosolic side to activate its RNase domain and triggers an unconventional splicing of the XBP1 mRNA. This splicing generates the transcription factor XBP1s which codes for the functionally active protein XBP1 [13]. Then, XBP1s promotes protein folding and induces endoplasmic reticulum stress-associated degradation (ERAD) via activating the transcription of genes [14]. IRE1α RNase can also cleave other ER-associated mRNA, degrading certain mRNAs through regulated IRE1-dependent decay (RIDD) and modulating diverse cellular responses [15]. Similarly, PERK undergoes oligomerization and auto-phosphorylation to phosphorylate the eukaryotic translation initiation factor eIF2α. This results in attenuation of the general translation of proteins while mediates the translation of specific mRNAs, such as that of the transcription factor ATF4 [16]. ATF6α is also another branch of the UPR. ATF6 activates after being transported from the endoplasmic reticulum to the Golgi apparatus where it undergoes S1P and S2P protease cleavage, releasing its cytosolic domain fragment which acts as a transcription factor.

## ERS responses in different molecular types of BC

The occurrence of different molecular types of BC are not equally distributed: ER+/PR+/HER2− (70% of patients), Her2+ (15%), and triple-negative BC (TNBC, 15%) [2, 17, 18]. In each of these different subtypes of BC, the role of ERS in progression varies [19].

### The role of ERS in estrogen receptor positive (ERα+) BC cells

Hormone receptor positive BC accounts for 65% of cases under 50 years old and 75% of cases in elderly women [20]. In hormone-sensitive BC cells, ERα is a proliferating factor which inhibits inflammatory responses, regulates lipid metabolism, and promotes the proliferation of tumor cells [21]. Under NCCN guidelines, adjuvant endocrine therapy is recommended for hormone receptor positive BC, including selective estrogen receptor modulator (SERM), selective estrogen receptor downregulator (SERD), aromatase inhibitor (AI), and GnHa. SERMs, such as tamoxifen, initiates cell apoptosis through over activation of nuclear ERα [22]. Recently, several studies have found that ERS in ERα+ BC could be divided into rapid UPR responded to ERα and long-term UPR responded to endocrine therapy [21, 23].

**Fig. 2:**
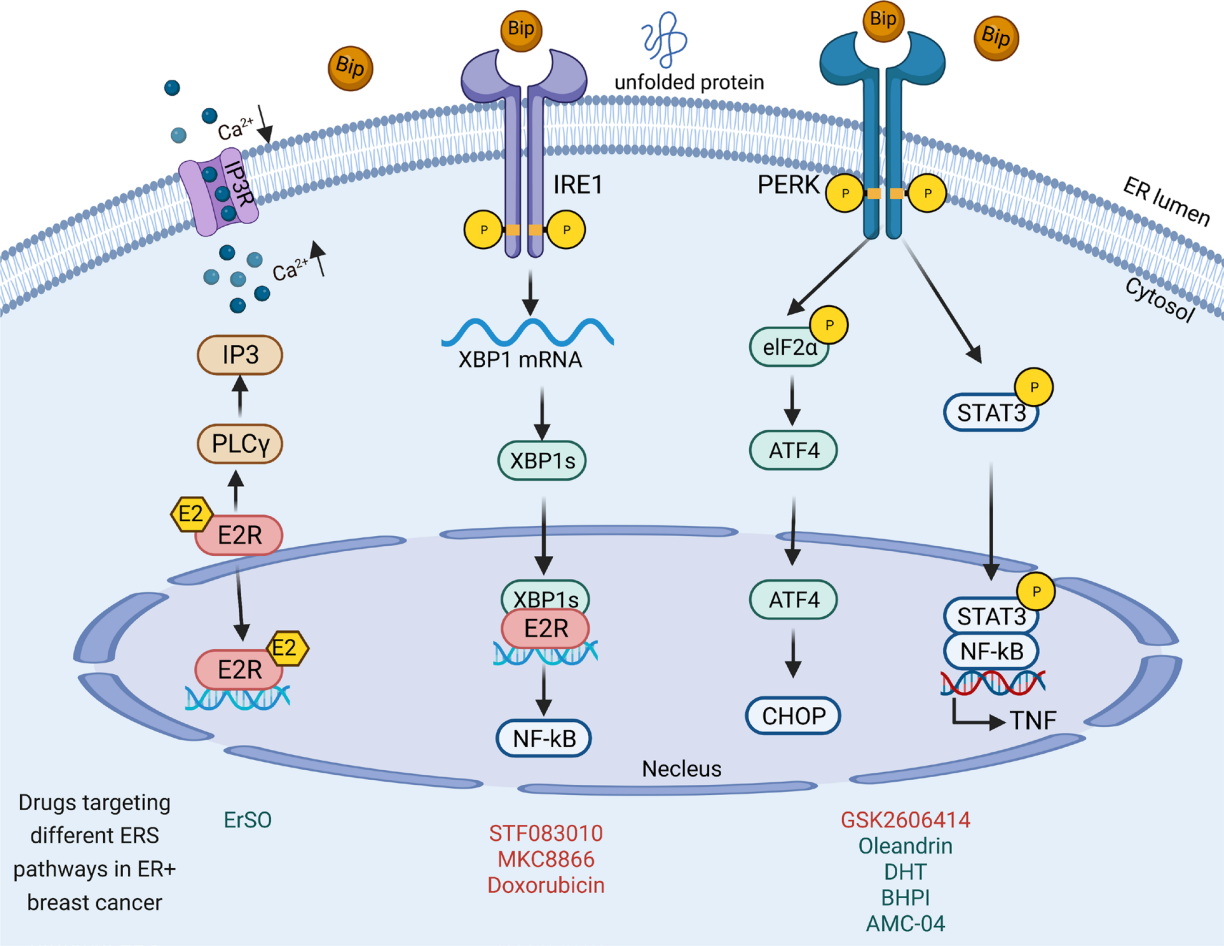
UPR Signaling in ERα+ BC. In ERα+ BC cells, ERα can open IP3R calcium channels through PLCγ activity and then induce rapid anticipatory activation of the UPR. The IRE1α-XBP1s pathway is activated to reestablish ER homeostasis. PERK-eIF2α-ATF4 can be activated to induce expression of apoptosis genes, such as CHOP. PERK can also be activated to promote apoptosis through TNFα expression promoted by NF-κB. The drugs targeting ERS-associated signaling pathways in ER+ BC are listed. Red represents pathway inhibitors and green represents pathway activators

As shown in Fig. 2, IRE1α-XBP1 signaling plays an essential role in the development of ERα+ BC. Estrogen (E2), acting via ERα, opens IP3R calcium channels mediated through a phospholipase Cγ (PLCγ) and then induces rapid anticipatory activation of the UPR [24]. XBP1 is expressed at high levels in ERα+ BC and co-expressed with ERα signaling [23, 25]. L. Wyld et al. found that the expression of XBP1 was noted in 90% of BCs and correlated with ERα+ (*P* = 0.017) by immuno-histochemical analysis of 395 BCs [26]. Moreover, ERα is not only the therapeutic target in ER positive BC patients, but also the site where take place of estrogen-induced endocrine resistance. Robert Clarke et al. found that XBP1s is involved in anti-estrogen resistance in protecting ERα+ BC by regulating NF-κB signaling [21, 27]. Thus, the estrogen signaling pathway and the IRE1-XBP1 axis generate a positive feed forward loop in BC [28].

**Fig. 3:**
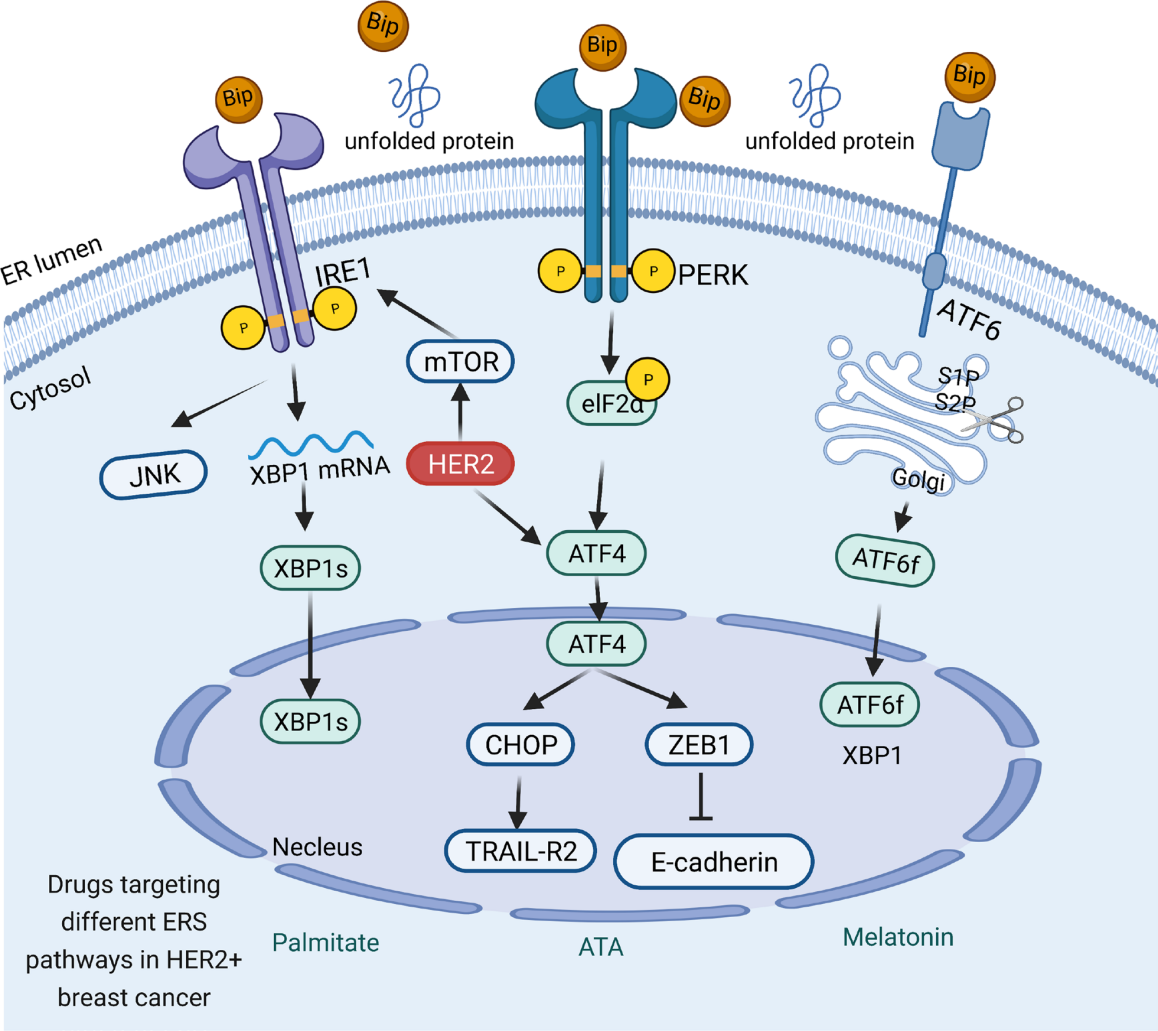
UPR Signaling in HER2+ BC. In HER2+ BC cells, HER2 amplification can activate the UPR through the PERK-ATF4-CHOP-TRAIL-R2 pathway, and the PERK-ATF4-ZEB1-E-cadherin pathway can regulate cell apoptosis and migration. Meanwhile, HER2-mTOR signaling can activate the IRE1 pathway. The drugs targeting ERS-associated signaling pathways in HER2+ BC are listed. Green represents pathway activators

In addition to IRE1α-XBP1 signaling, PERK also plays a key role in ERα+ BC. In addition to routine attenuation of protein translation through phosphorylated eIF2α, PERK was also persistently activated by ERα and then induced activation of ATF4 and CHOP [29]. Jordan VC et al. found that the PERK-NF-κB-TNFα axis could mediate estrogen-induced apoptosis [30]. Also, estrogen was shown to promote ERα+ BC by inducing the expression of UPR regulator GRP78 [26, 31]. GRP78 could activate all branches of UPR transducers and contributed to promote cell survival and proliferation [24]. Therefore, ERα induces the UPR in sensitive and hormone-resistant BC cells with varying consequences, depending on the duration and intensity of the stress (Fig. 3).

### The role of ERS in HER2+ BC cells

HER2 is a receptor tyrosine kinase amplified and overexpressed in 15–20% of BCs, which is associated with an aggressive clinical course and early metastasis. Several investigations have revealed the sensitivity of HER2 to agents that induce the UPR in BC cells [32, 33]. For example, Abelardo et al. found that HER2 had increased sensitivity to ER stress through the PERK-ATF4-CHOP pathway, resulting in the upregulation of the pro-apoptotic cell surface receptor TRAIL-R2 and activated caspase-8 [33]. Chen s et al. found that HER2 upregulated ATF4 expression to improve ZEB1 and suppress E-cadherin, resulting in increased cell migration [34]. Meanwhile, HER2-mTOR signaling-driven BC cells require ER-associated degradation for survival [35]. Wegwitz et al. found that USP22 actively suppressed UPR induction in HER2 BC by stabilizing the major ER chaperone HSPA5 [36]. Also, Maurizio et al. found that activation of the UPR bypassed trastuzumab-mediated inhibition of the PI3K/AKT pathway [37]. Thus, selectively targeting ERS pathways in combination with HER2-targeting agents may have therapeutic benefits in the treatment of HER2 positive BC.

### The role of ERS in TNBC BC cells

Triple-negative BC (TNBC) lacks targeted therapies and has the poorest outcomes when compared to the other types of BC [38]. Similar to what is observed in ERα+ BC, the majority of TNBC patients tend to develop some degree of drug resistance [39].

The mechanisms of ERS in TNBC were related with the three branches of ERS. Laurie et al. found that the transcriptional activity of XBP1 was activated in TNBC but not in ER+ BC. XBP1s was shown to interact with HIF1α, creating an XBP1s–HIF1α complex via the recruitment of RNA polymerase II, which promoted TNBC development and poor prognosis [40]. These reports suggest that XBP1 is overexpressed in luminal cancers while increased XBP1s transcriptional activity is more strongly associated with TNBC. Thus, TNBC cells critically rely on IRE1α to adapt ERS and adjust the tumor microenvironment (TME) to facilitate malignant growth [41]. In addition, IRE1α–XBP1s pathway can be activated by c-MYC in TNBC and then sustain cell growth and survival [42]. Meanwhile, the PERK–eIF2α pathway induces autophagy and redox control in TNBC. Lopez et al. found that caspase-8 and Noxa-activated apoptotic mechanisms are activated in TNBC cells undergoing sustained ERS [43]. ERS inhibits androgen receptor (AR) expression via the PERK-eIF2α-ATF4 pathway [44].

**Fig. 4:**
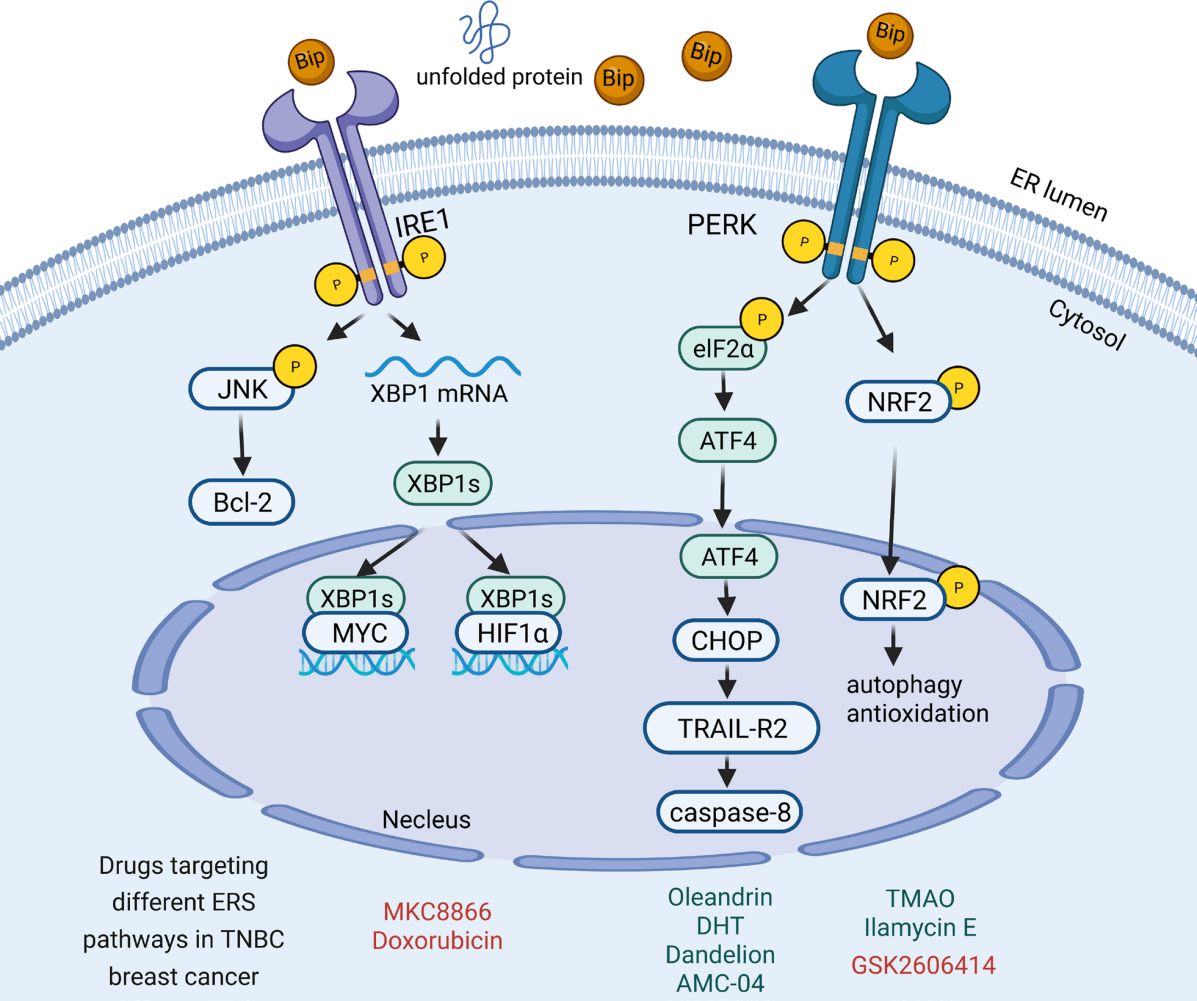
UPR Signaling in TNBC. In TNBC cells, the IRE1α–XBP1s pathway can interact with HIF1α and c-MYC to participate in cell survival, angiogenesis, and invasion. Meanwhile, the PERK–eIF2α pathway activates either the peIF2α–ATF4 pathway or the transcription factor NRF2 to induce autophagy and redox control. The drugs targeting ERS-associated signaling pathways in TNBC are listed. Red represents pathway inhibitors and green represents pathway activators

Other indirect mechanisms activating ERS could function in TNBC. ER-oxidoreductase 1α (ERO1α) is an oxidase located in the ER which controls oxidative protein folding. ERO1α was reported to be upregulated in BC and is correlated with poor recurrence-free survival in TNBC [45]. ERO1α promotes angiogenesis by increasing VEGF expression and promoting immune escape via PD-L1 and chemokines in TNBC [46–48]. Moreover, Wood et al. found that insulin-like growth factor type 1 receptor (IGF-1R) inhibition promoted TNBC by increasing ROS-mediated ERS [49]. Also, ERS induced CHOP and JNK pathways, which are known to play an important role in TNBC [50, 51]. Thus, different ERS mechanisms in TNBC could represent critical treatment targets (Fig. 4).

## Drugs targeting ERS-associated signaling pathways in BC

Cancer cells rely on high levels of ER stress response to deal with misfolded proteins and rapid growth. Excessive and prolonged UPR activation has been shown to promote the resistance of BC cells to chemotherapy and radiotherapy [52]. Therefore, altering UPR signaling to disrupt this balance between surviving ER stress and UPR-initiated apoptosis could effectively induce cell death in BC cells. Recently, some drugs that promote BC cell sensitivity to treatment have been identified as having effects on UPR signaling. For example, doxorubicin had been identified as a novel inhibitor of the IRE1α-XBP1 pathway, which was previously unknown [53]. Thus, we summarized the different drugs targeting ERS-associated signaling pathways in the treatment of BC. The drugs which can target ERS and the UPR to treat BC are summarized in Table 1.

**Table 1:** Drugs targeting ERS to treat breast cancer

Drugs	Mechanism	Effect	Refs
Targeting IRE1α-XBP1 signaling			
MKC8866	Selective IRE1 RNase inhibitor	MYC-driven BC apoptosis	[42]
STF083010	XBP1 splicing inhibitor	Improve tamoxifen sensitivity	[57]
Doxorubicin	IRE1α-XBP1 inhibitor	Cell apoptosis	[53]
Palmitate	IRE1-mediated XBP1 splicing activator	Improve trastuzumab sensitivity in HER2+ BC	[96]
Targeting PERK-elF2α-ATF4 signaling			
GSK2606414	PERK inhibitor	Improve radiotherapy sensitivity	[62]
Oleandrin	p-PERK activator	Trigger immunogenic cell death (ICD)	[63]
DHT	p-PERK activator	Cell apoptosis	[64]
BHPI	Persistent ERα- dependent PERK activator	Endocrine-resistant BC apoptosis and necrosis	[65]
Dandelion extract	p-PERK activator	TNBC cell apoptosis	[97]
Ilamycin E	Trigger CHOP/Bcl-2	TNBC cell apoptosis	[50]
TMAO	PERK activator	Promote immunotherapy in TNBC	[95]
AMC-04	Activate ATF4/CHOP/DR5	Cell apoptosis	[66]
ATA	PERK activator	HER2 + BC cell apoptosis	[98]
ISRIB	eIF2α inhibitor	Prevent breast cancer cells with stem-cell-like features (BCSC)	[69]
Targeting GRP78			
HHQ-4	GRP78 inhibitor	Glucose-deprived BC cells apoptosis	[74]
Plumbagin	GRP78 inhibitor	Improve tamoxifen sensitivity	[75]
Kaempferol	GRP78 inhibitor	Cell apoptosis	[76]
Epigallocatechin gallate	GRP78 inhibitor	Improve taxol and vinblastine sensitivity	[91]
JI017	GRP78 activator	Improve paclitaxel sensitivity	[77]
Betulinic acid	GRP78 activator	Improve taxol sensitivity	[92]
Prodigiosin	GRP78 activator	Cell apoptosis	[78]
Tunicamycin	GRP78 activator	Cell apoptosis	[79]
Panobinostat	GRP78 hyperacetylation	Cell apoptosis	[80]
Targeting other pathways			
Melatonin	Promote ROS	Improve lapatinib sensitivity in HER2+ BC cells	[81]
DK143	Promote ROS	Cell apoptosis	[82]
ErSO	Open IP3R calcium channels	Induce rapid and selective necrosis of ERα+ BC	[83]
MPPa-PDT	Induce autophagy	Cell apoptosis	[84]

### Drugs targeting IRE1α-XBP1 signaling

Several studies have developed various specific inhibitors for different components of the IRE1α-XBP1 signaling pathway. Inhibitors targeting IRE1α-XBP1 include two drugs targeting RNase activity and protein kinase activity. Several IRE1 RNase inhibitors have shown efficacy in in vivo models of multiple myeloma, such as MKC3946 and STF083010 [54, 55]. Treatment efficacy with these inhibitors was unclear in the treatment of solid tumors.

MKC8866, as a selective IRE1 RNase inhibitor, can decrease the production of cytokines, including IL-6, IL-8, and TGFβ, and promote paclitaxel sensitivity in TNBC [56]. Zhao et al. also found that the MKC8866 inhibition of the IRE1–XBP1 pathway can suppress MYC-driven BCs [42]. STF083010, an inhibitor that specifically blocks XBP1 splicing, was able to re-established tamoxifen sensitivity in resistant MCF-7 cells [57].

### Drugs targeting PERK signaling

There are several ATP-competitive PERK kinase inhibitors, such as GSK2606414 and GSK2656157 [58, 59]. GSK2606414 has been found in the treatment of several cancers such as head and neck squamous cell carcinoma and pancreatic cancer [58, 60]. GSK2656157 has been found in the treatment of lung cancer and esophageal squamous carcinoma [59, 61]. Span et al. also found that PERK inhibitor GSK2606414 could improve radiotherapy sensitivity in BC cells [62].

Conversely, Overstimulation of the PERK pathway effectively induces cancer cell apoptosis, likely through pro-apoptotic effects of CHOP. For example, Oleandrin, a cardiac glycoside, can induce ERS-associated, caspase-independent ICD in BC cells through the PERK-elF2α-ATF4-CHOP pathway [63]. Dihydrotanshinone I (DHT) has been shown to activate the PER-elF2α-ATF4 pathway and which then triggered BC cell apoptosis [64]. Also, Shapiro et al. reported that ERα biomodulator BHPI induces persistent ERα-dependent PERK activation which promotes apoptosis and necrosis in endocrine-resistant BC cells [65]. Inki Kim et al. found a new piperazine oxalate derivate (AMC-04) that induces apoptosis via activation of the ATF4/CHOP/DR5 pathway [66].

Moreover, an integrated stress response (ISR), which aimed to restore cellular homeostasis, promoted phosphorylation of eIF2α [7, 67]. As an important eIF2α inhibitor, ISR inhibitor (ISRIB) was found to inhibit eIF2α phosphorylation by activating eIF2B, thus inhibiting signaling downstream from eIF2α to ATF4 [68]. In BC, Michael Jewer et al. (year) found that ISRIB can effectively prevent phenotypes of BC cells that have stem-cell-like features (BCSC) and improve outcomes with mTOR inhibitors or chemotherapy [69]. Lee et al. (year) also found that ISRIB combined with bortezomib could trigger paraptosis in BC cells [70].

### Drugs activating ATF6

Unlike IRE1 and PERK, few selective modulation agents of ATF6 had been developed. The inhibitions of ATF6 such as ceapins were achieved by inhibiting the proteases from S1P and S2P [71]. Also, protein disulfide isomerase (PDI) contributed to disulfide bond rearrangement in ATF6 under stress conditions and several studies found PDI inhibitors such as 16F16 and P1 [72, 73]. However, in BC, none inhibitors directly targeting ATF6 had been found.

### Drugs targeting GPR78

GRP78 controls the activation of endoplasmic reticulum-transmembrane signaling mechanisms. HHQ-4 is a quinoline derivate and GRP78 inhibitor that preferentially inhibits proliferation of glucose-deprived BC cells [74]. Plumbagin, another GRP78 inhibitor, was able to sensitize BC cells to undergoing tamoxifen-induced cell death [75]. Also, Ravanan et al. found that GRP78 inhibitor kaempferol could induce cell death by targeting CHOP and caspase 3/7 [76].

Moreover, except GRP78 inhibitors, there are some ERS inducers activating GRP78. For example, Seong-Gyu Ko et al. developed a novel herbal extract called JI017, which can activate GRP78 from both exosomes and cell lysates to induce an excessive UPR in paclitaxel-resistant BC [77]. Ewa et al. found that prodigiosin could upregulate GRP78 and then induce both IRE1–JNK and PERK–eIF2α signaling pathways, which were essential to upregulate CHOP and suppress BCL2 to evoke cell death [78]. Tunicamycin was found to not only increase GRP78 expression in ER-/PR-/HER2+ BC, but also in ER-/PR-/HER2- BC, which was associated with high anti-tumorigenic action [79].

Almost all the drugs targeting GRP78 were targeting the protein expression level of GRP78. Recently, a regulator targeting GRP78’s post-translational modifications was found. For example, Balusu et al. found that panobinostat, a pan-histone deacetylase (HDAC) inhibitor, was able to bind and hyperacetylate GRP78, which then then activated the PERK-elF2α-CHOP pathway to induce cell death [80].

### Indirect activation of UPR signaling by small molecule therapy

Outside of the three main branches of the UPR, some drugs can indirectly activate the UPR through other mechanisms. For example, melatonin can enhance the cytotoxic effect of lapatinib in HER2-positive BC by inducting ER stress through promoting excessive UPR and ROS accumulation [81]. Soon Young Shin et al. found that the synthetic chalcone derivative DK143 can be used to promote BC apoptosis by inducing ROS-mediated activation of the UPR [82].

Moreover, David J. Shapiro et al. discovered the compound ErSO, which activates the anticipatory UPR by promoting a rapid efflux of calcium stored in the ER into the cytosol, which induces rapid and selective necrosis of ERα-positive BC cells in a patient-derived xenograft (PDX) mouse model [83]. Also, ErSO treatment induced XBP1s mRNA > 1,000 fold higher than the previously reported activator BHPI, converting the UPR from protective to toxic by opening ER IP3R calcium channels [65]. Bai DQ et al. found that methyl pyropheophenylchlorin photodynamic therapy (MPPa-PDT) can inhibit tumor growth through ERS-induced autophagy in vitro and in vivo [84].

## Discussion

Over the past decades, significant discoveries have helped establish ERS and UPR as the protein homeostasis regulation mechanisms, which balance survival and progression of tumor cells [7]. While extensive research has focused on characterizing and modulating ERS between healthy and disease states, little is known about the role and clinical application of ERS in certain cancers due to the tumor specificity. In particular, several questions remain unresolved. How does ERS affect different molecular types of BC cells? How do ERS-targeting drugs impact BC cells? How can other forms of cancer therapy, particularly immunotherapy, be combined with ERS-targeting drugs? To answer these questions, it is critical to gain a comprehensive understanding of the mechanisms and clinical applications of ERS in BC. In this review, we summarized the main functions and mechanisms of ERS in different molecular types of BC and focused on drugs that have potential for targeting ERS in the treatment of BC.

The role of ERS in BC depends on its molecular types. Several studies have shown that ERS responses were employed by estrogen to regulate the development of BC cells [24]. ERα and its agonists activated the IRE1, PERK, and ATF6 pathways. Also, over activating the pro-apoptotic branches of the UPR could activate a ligand-independent apoptotic program in HER2+ BC cells [33]. Laurie et al. showed that total XBP1 was overexpressed in luminal cancers while increased XBP1s transcriptional activity was more strongly associated with TNBC [40]. In addition to directly regulating cancer characteristics, ERS can be transmitted to and dynamically reprogram tumor-infiltrating immune cells, especially myeloid cells [85–87]. For example, ERS has been shown to promote macrophage activation and induce pro-inflammatory responses [88]. Also, ERS can disrupt the metabolism and antigen presenting capacity of dendritic cells (DCs) and inhibit T cell proliferation [88, 89]. Moreover, GRP78 and CD47 co-expression results in increased tumor macrophage infiltration and is associated with poor prognosis in BC patients [90]. Therefore, drugs targeting ERS and its downstream signaling pathways are essential in stopping tumor growth, metastasis, and improving responses to chemotherapy, targeted therapy, and immunotherapy.

In summary, ERS related antitumor drugs can be divided into those that inhibit UPR-mediated survival and those that induce sustained ERS-mediated death. ERS-targeting drugs can not only directly promote apoptosis of BC cells, but also enhance the effect of traditional treatment. STF083010 and Plumbagin have been shown to promote sensitivity to tamoxifen in BC cells [57, 75]. Epigallocatechin gallate and betulinic acid can improve the sensitivity of BC cells to the chemotherapy taxol [91, 92]. PERK inhibitor GSK2606414 can improve radiotherapy sensitivity [62]. Also, due to the function of ERS in immune cells, targeting ERS can be a new strategy for immune modulation and immunotherapy in BC treatment [90, 93]. Cubillos-Ruiz et al. found that the upregulation of XBP1 was related to a decrease in T cell infiltration [94]. TMAO has been identified as a drug that helps promote the efficacy of immunotherapy in TNBC treatment [95]. Also, Oleandrin can trigger ICD by activating p-PERK [63].

Although research on drugs targeting ERS has made significant progress, some problems remain unsolved. For example, IRE1 inhibitors can produce undesired side effects due to blocking XBP1s and increasing unspliced XBP1, which could lead to increased NF-κB in ER-positive BC [27]. Thus, it is important to develop new strategies to directly target XBP1. Also, PERK inhibitor GSK2606414 had serious toxic side effect on the pancreas and significantly inhibited the production of insulin [62]. Thus, further preclinical and clinical studies are necessary to evaluate their anti-tumor efficacy and potential side effects in combination with other forms of therapy.

In conclusion, ERS and UPR signaling is involved in promoting the development and progression of certain types of BC, as well as contributing to therapy resistance. The significance of the UPR is specific to different molecular types of BC. Several studies identified that drugs targeting the underlying mechanisms driving the UPR improve treatment outcomes in BC patients. In the future, more clinical trials are needed to verify the efficacy of these specific anti-UPR drugs for the treatment of BC.

## Data Availability

Not applicable.

## Abbreviations

BC: Breast cancer
ERS: Endoplasmic reticulum stress
UPR: Unfolded protein response
ERα/ESR1: Estrogen receptor
PGR: Progesterone receptor
ERBB2/Her2: Human epidermal growth factor receptor 2
TNBC: Triple-negative breast cancer
ICD: Immunogenic cell death
IRE1: Inositol requiring enzyme 1
PERK: Protein kinase R-like ER kinase
ATF6: Activating transcription factor 6
GRP78/BiP: 78-KD glucose-regulated protein
ERAD: Endoplasmic reticulum stress associated degradation
RIDD: Regulated IRE1-dependent decay
SERM: Selective estrogen receptor modulator
SERD: Selective estrogen receptor downregulator
AI: Aromatase inhibitor
TME: Tumor microenvironment
AR: Androgen receptor
IGF-1R: Insulin-like growth factor type 1 receptor
DHT: Dihydrotanshinone I
AMC-04: Piperazine oxalate derivate
PDI: Protein disulfide isomerase
HDAC: Histone deacetylase
PDX: Patient-derived xenograft
